# Need for Improved Risk Communication of Fish Consumption Advisories to Protect Maternal and Child Health: Influence of Primary Informants

**DOI:** 10.3390/ijerph10051720

**Published:** 2013-04-29

**Authors:** Catherine E. LePrevost, Kathleen M. Gray, Mercedes Hernández-Pelletier, Brennan D. Bouma, Consuelo Arellano, W. Gregory Cope

**Affiliations:** 1 Department of Environmental and Molecular Toxicology, North Carolina State University, Box 7633, Raleigh, NC 27695, USA; E-Mails: greg_cope@ncsu.edu; 2 UNC Institute for the Environment, University of North Carolina at Chapel Hill, 137 E. Franklin Street, Suite 602, Chapel Hill, NC 27599, USA; E-Mails: kathleen_gray@unc.edu (K.M.G.); bbouma@tjcog.org (B.D.B.); 3 Occupational and Environmental Epidemiology Branch, Division of Public Health, North Carolina Department of Health and Human Services, 1912 Mail Service Center Raleigh, NC 27699, USA; E-Mail: mercedes.hernandez-pelletier@dhhs.nc.gov; 4 Department of Statistics, North Carolina State University, Box 8203, Raleigh, NC 27695, USA; E-Mail: arellano@stat.ncsu.edu

**Keywords:** fish consumption, communication, fish advisory, pregnant women, children

## Abstract

Fish consumption has established benefits, including the promotion of cardiovascular health and pre- and neonatal brain and eye development, but local freshwater fish may be a source of contaminants that are especially harmful to fetuses and young children, such as the neurotoxic and developmentally toxic methylmercury and polychlorinated biphenyls. Fish consumption advisories may be issued by state health departments to limit human exposure to these and other toxicants. This study examined the efficacy of a sign designed by the North Carolina Division of Public Health that was posted along a reservoir (Badin Lake) in central North Carolina, USA, for increasing anglers’ awareness of a fish consumption advisory, with a special focus on anglers who share their catch with women and children. In this study, 109 anglers were interviewed about their awareness of fish consumption advisories in general and their knowledge of the Badin Lake fish advisory in particular. Shore anglers were significantly less likely to be aware of the term “fish consumption advisory” and of the specific advisory for Badin Lake than boat anglers. Although a significant increase in knowledge of the specific fish consumption advisory was found for the entire sample of study participants after the sign intervention, a commensurate increase in knowledge was not found for a subsample of anglers who reported sharing their catch with women and children. Study findings underscore differences in fish consumption advisory awareness among subpopulations. Specifically, the study revealed the importance of characterizing the communication needs of shore anglers and anglers who share their catch with sensitive subpopulations (e.g., women and children) for the creation of more targeted communications of fish consumption advisories.

## 1. Introduction

### 1.1. Fish Consumption: A Public Health Issue for Women of Childbearing Age and Children

Recent review articles have highlighted the need for consumers, particularly women of childbearing age and children, and those providing dietary advice or public health recommendations to these groups to weigh benefits and risks of fish consumption [1,2,3]. Health benefits ascribed to fish consumption for the general population and for these sensitive subpopulations relate to the association of n-3 polyunsaturated fatty acids (omega-3 fatty acids) with cardiovascular health and pre- and neonatal brain and eye development. Contaminants in fish, specifically methylmercury and polychlorinated biphenyls (PCBs), however, pose health risks that are of particular concern for fetuses and young children due to their neurotoxic and developmentally toxic effects and the propensity for these chemicals to cross the placenta and to contaminate breast milk [4]. 

Consumption of fish highly contaminated with methylmercury has been associated with permanent nervous system damage and with developmental delays, ataxia, blindness, mental retardation, and spasticity in the children of mothers with high-level methylmercury exposure during pregnancy or while breast feeding [5,6]. The historical case of Minamata disease in the 1950s and 1960s on Japan’s Kyushu Island was important in elucidating that high doses of methylmercury that resulted in minor symptoms in mothers could be extremely toxic to fetuses. Children whose mothers consumed fish and shellfish contaminated by methylmercury in waste water discharged from a chemical plant presented with symptoms of cerebral palsy (including mental retardation, ataxia, hyperkinesia, and dysarthria) [7]. Recent fish methylmercury data suggest that recreationally caught fish in lakes of the United States (U.S.) are substantially less contaminated than those described in the Minamata case, but contamination levels are still of concern [8]. 

PCB exposure in pregnant women has been associated with deficits in motor skills and short-term memory in infants [9]. PCBs are most effectively eliminated from mothers’ bodies through breast milk, and contaminated breast milk is a considerable source of infant exposure [10]. In addition, Newland [4] suggests that methylmercury and PCBs might interact synergistically, which is important because these contaminants often coexist in fish and result in co-exposure in humans. 

In response to the health risks associated with methylmercury and the ubiquitous contamination of fish and shellfish with mercury, the U.S. Environmental Protection Agency and the U.S. Food and Drug Administration advise women who may become pregnant, pregnant women, nursing mothers, and young children to avoid certain fish species high in methylmercury, to eat up to two meals (12 ounces uncooked weight) weekly of fish species lower in mercury, and to consume up to one meal (6 ounces uncooked weight) weekly of fish caught in local waters when no local fish consumption advisory exists [11]. More restrictive consumption advisories may be in effect locally for particular species of fish and bodies of water for chemical contaminants of concern, including methylmercury and PCBs. According to Ney and Ney [12], the issuance of fish consumption advisories has increased over the past decade, with methylmercury and PCB contamination accounting for more than 80% of existing advisories and with women of childbearing age and children comprising the most sensitive target group for such advisories.

### 1.2. Study Context

Badin Lake is a 5,300-acre reservoir on the Yadkin-Pee Dee River located in central North Carolina, USA that is used for fishing, boating, and swimming. An estimated 9,550 persons annually engage in recreational fishing from the two counties (Stanly and Montgomery) adjacent to Badin Lake [13], with many unquantified others coming from the surrounding region. Angler effort and harvest have traditionally been the greatest for the species crappie (*Pomoxis* spp.), sunfish (*Lepomis* spp.), catfish (*Ictalurus* spp., *Ameiurus* spp., *Pylodictis olivaris*), and largemouth bass (*Micropterus salmoides*) [14]. 

In 2008, in response to concerns at the county level regarding elevated PCB concentrations in sediment and fish samples, the local health departments asked the North Carolina Division of Public Health to conduct an evaluation of the potential for people to be exposed to PCBs as a result of consuming fish from Badin Lake. Prior sediment samples associated with Resource Conservation and Recovery Act (RCRA) facility investigations had measured PCB concentrations in the lake [13]. Sampling of fish and a thorough public health assessment were conducted in 2008. The findings resulted in the State of North Carolina issuing a fish advisory for PCBs in Badin Lake in early 2009. The fish consumption advisory recommends that pregnant women, women who may become pregnant, nursing mothers, and children under the age of 15 consume no catfish or largemouth bass from Badin Lake and that all others eat no more than one meal a week of these fish. At the time that the advisory was issued for PCBs, Badin Lake was already included in a statewide advisory for methylmercury. This pre-existing advisory recommends that women of childbearing age, pregnant women, nursing mothers, and children under the age of 15 do not eat fish high in mercury caught in the state, including largemouth bass, and eat up to two meals per week of fish low in mercury. Other individuals are recommended to eat no more than one meal per week of fish high in mercury and four meals per week of fish low in mercury. 

**Figure 1:**
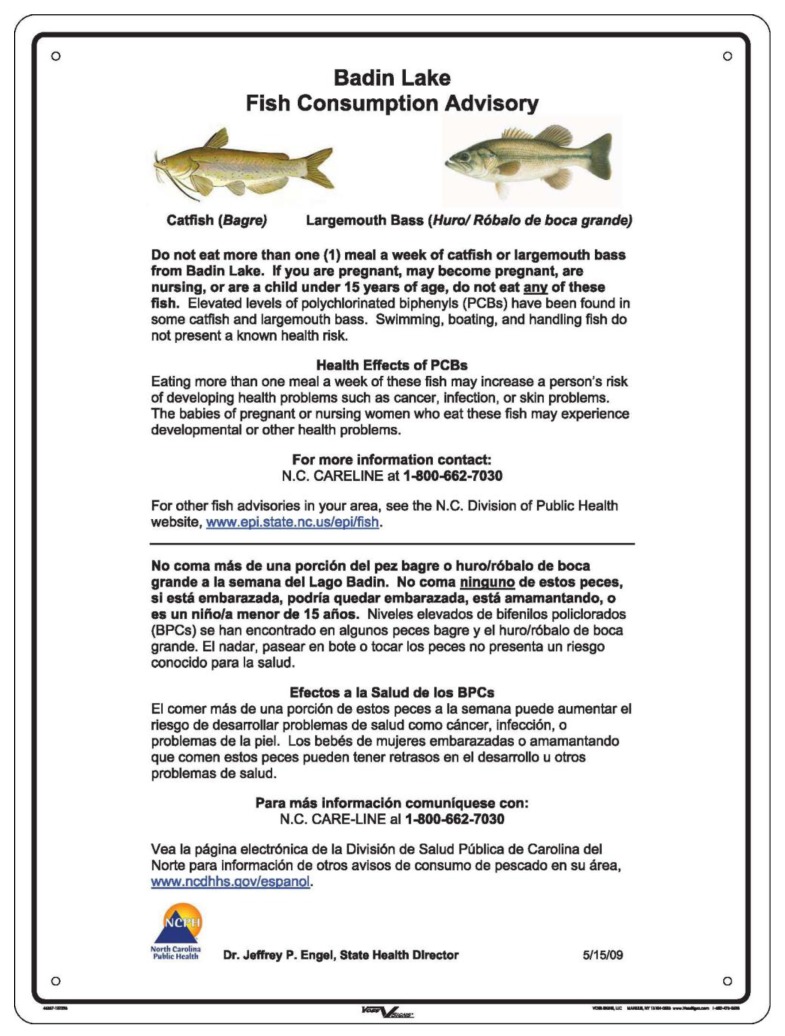
Informational sign designed by the North Carolina Division of Public Health to communicate the fish consumption advisory at Badin Lake, NC, USA. (Courtesy of the North Carolina Department of Health and Human Services)

The PCB fish advisory was issued on 11 February 2009, and informational signs (Figure 1) were installed on 21 August 2009, after the coordination between the two local health departments to find resources for the design, production, and deployment of signs. Figure 1 depicting the sign was provided by the North Carolina Department of Health and Human Services, which designed and printed the signs with financial resources from the Stanly and Montgomery County Health Departments. Twenty-five metal signs and metal posts were installed at primary access points around the lake by staff of the North Carolina Wildlife Resources Commission. Limited resources and the need for the advisory to be communicated immediately to Badin Lake anglers prohibited the development of research-based signage designed with input from the end-user. The purpose of this study, therefore, was to assess the efficacy of the signs in increasing angler awareness of the fish advisory, particularly the awareness of anglers who share their catch with the most sensitive subpopulations. 

### 1.3. Research Question

This study engaged anglers in interviews about their awareness of fish consumption advisories generally and about their knowledge of the Badin Lake fish advisory in particular. The following research question guided this study: Do the signs increase anglers’ awareness of the fish consumption advisory in effect at Badin Lake? Also of interest was determining if sensitive subpopulations were adequately informed.

## 2. Methods

A pre- and post-intervention study design was implemented in this study. A convenience sample of 109 anglers participated in interviews, with 56 participants involved in the pre-intervention stage and 53 in the post-intervention stage. Participant interviews occurred before and after fish consumption advisory signs were posted at Badin Lake to determine if the signs increased awareness of the advisory in effect. In all, 25 signs (18″ × 24″ inches in size; Figure 1) were posted in 17 areas (including boat launches, fishing piers, picnic areas, swimming areas, and campgrounds) along the shore in the two counties adjacent to Badin Lake. 

### 2.1. Interview Guides

Two interview guides were developed for this study, one for pre-intervention interviews and one for post-intervention interviews. Interview guides included closed-ended items designed for oral administration in English and Spanish. The decision to conduct interviews in English and Spanish was based on information about the Badin Lake community provided by the local health departments’ staff. Twelve items were included on the pre-intervention interview guide, and 15 items were included on the post-intervention interview guide. Items related to demographics (gender, age, and educational attainment), fishing habits, fish eating habits, sharing fish with women and children, knowledge of the term “fish consumption advisory”, and knowledge of the fish consumption advisory in effect for Badin Lake. The post-intervention interview guide additionally included items pertaining to the signs posted at Badin Lake and an open-ended comment item related to impressions and preferences. The interview guide was developed and pre-tested by the authors and knowledgeable state public health officials and was evaluated with members of a local angling population. The interview methodology and guides were reviewed and approved (exempt status) by the Division of Public Health’s Institutional Review Board.

### 2.2. Interviews

Study participants were interviewed while they were fishing at Badin Lake at various locations within the area between the Tuckertown Dam (most upstream location) and the Narrows Dam (most downstream location). Interviews were conducted in English or Spanish, depending on participant preference. Potential participants were asked if they fished at the lake, if they were 18 years of age or older, and if they would be willing to participate in a short survey. Participants were selected for participation in interviews only if they answered affirmatively to all three of these questions. 

Interviews were conducted over a span of four days, all Saturdays, when the greatest angling effort presumably occurred. Two interview days occurred before the signs were installed, and two interview days occurred after the signs were installed. The pre-intervention interview guide was used for interviews conducted on 1 and 15 August 2009, prior to the installation of the signs. The post-intervention interview guide was implemented on 14 November 2009, and 10 April 2010, after the signs were installed. Interviews were 5 to 15 min in duration. The interviews were conducted over the entire day for the four interview days, starting at sunrise, ending at sunset, and rotating hourly among 17 sign locations in a pre-determined manner. Interview locations were the same in pre- and post-intervention interviews and were selected according to public access points, such as boat launches and parks. An informal training was provided to each interviewer before s/he conducted any interview. The training consisted of background information about the fish study at Badin Lake, fish advisory details, how to approach the interviewee, how to ask each question, and how to record the responses. One single author was present during all interviews, and the first interview of each day was conducted jointly by the interview team.

### 2.3. Data Analysis

Statistical analyses were performed separately for the entire sample of anglers (n = 109) and for the subsample of anglers who indicated that they share the fish that they catch at Badin Lake with women and/or children (n = 63). *p*-values of <0.05 were considered significant, and *p*-values reported for pairs of ordinal variables are for one-sided Fisher’s Exact tests. These data analyses were conducted with Epi Info software, version 3.5.1 (U.S. Centers for Disease Control and Prevention, Atlanta, GA, USA). The Cochran-Mantel-Haenszel (CMH) correlation statistic was used to test whether there was a relationship between two ordinal variables after blocking across a third classification variable, as a way to adjust by its confounding effect. These analyses were conducted with a non-significant CMH statistic SAS version 9.2 Procedure FREQ with Tables Statement and Option CMH for calculation of the generalized CMH statistic (SAS Institute Inc., Cary, NC, USA).

## 3. Results

### 3.1. Participant Description

Table 1 presents demographic information on the overall sample population and the subsample of interest that shares fish caught at Badin Lake with women and/or children. Pre- and post-intervention samples were demographically similar. 

**Table 1:** Demographic characteristics of overall sample of fish consumption advisory study participants at Badin Lake, NC, USA compared to a subsample that shares fish caught with women and children.

Category	Overall	Sharers
*Gender*		
Male	81% (n = 88)	84% (n = 53)
Female	19% (n = 21)	16% (n = 10)
*Age (years)*		
18–24	5% (n = 6)	6% (n = 4)
25–34	25% (n = 27)	27% (n = 17)
35–44	21% (n = 23)	22% (n = 14)
45–54	23% (n = 25)	19% (n = 12)
55 and older	26% (n = 28)	26% (n = 16)
*Highest Educational Attainment*		
Grades 1–6	6% (n = 6)	10% (n = 6)
Grades 6–9	13% (n = 14)	15% (n = 9)
Grades 10–12	45% (n = 49)	45% (n = 28)
College	32% (n = 35)	29% (n = 18)
Graduate School	4% (n = 4)	1% (n = 2)
*Fishing Location*		
Boat	45% (n = 49)	38% (n = 24)
Shore	55% (n = 60)	62% (n = 39)
*Frequency of Fishing*		
Less than 1 time per month	15% (n = 16)	16% (n = 10)
1 time per month	20% (n = 22)	16% (n = 10)
1–2 times per week	48% (n = 52)	57% (n = 35)
More than 3 times per week	17% (n = 18)	11% (n = 7)

### 3.2. Anglers Sharing with Women and Children

Table 2, Table 3 present group differences in knowledge of the term “fish consumption advisory” and knowledge of the advisory in effect for Badin Lake, respectively, for the overall sample of Badin Lake anglers and for the subsample of interest that shares fish caught at Badin Lake with women and/or children. Sixty-seven of the anglers in the sample (61%) reported that they eat the fish they catch at Badin Lake. Of the 67 participants who indicated that they consume the fish that they catch in Badin Lake, 63 (94%) reported sharing the fish with women and/or children. 

For the overall sample, increasing age generally corresponded to an increase in knowledge of the “fish consumption advisory” term, but not at a statistically significant level (*p* = 0.0920). Knowledge of the Badin Lake-specific advisory significantly increased with age for the overall sample (*p* < 0.001) and for the sharing subsample (*p =* 0.0131). The relationship between knowledge of the Badin Lake advisory and age remained significant (*p* = 0.0006 for overall sample; *p =* 0.0112 for sharing subsample) after controlling for education level. Although no significant relationship was observed between knowledge of the Badin Lake advisory and consumption of fish from Badin Lake, the percentage of anglers who did not know the term “fish consumption advisory” was greater (*p =* 0. 0328) among those who consume fish (51%) than those who do not (31%). Knowledge of the term “fish consumption advisory” varied significantly by education level for all anglers (*p* < 0.001) and for the sharing subsample (*p* = 0.0019). Increased knowledge was observed among anglers with higher education levels.

**Table 2:** Group differences in knowledge of the term “fish consumption advisory” for the overall sample of study participants at Badin Lake, NC, USA compared to a subsample that shares fish caught with women and children.

Variable of Comparison	Knowledge by Comparison Groups					*p-*value *	*p-*value Controlling for Education ^†^
***Age (years)***	***18–24***	***25–34***	***35–44***	***45–54***	***55+***		
Overall	50% (n = 3) ^††^	48% (n = 13)	48% (n = 11)	64% (n = 16)	68% (n = 19)	0.0920	0.0701
Sharers	50% (n = 2)	41% (n = 7)	50% (n = 7)	50% (n = 6)	56% (n = 9)	0.4972	0.4003
***Consumption of Fish from Badin Lake***	***No***	***Yes***					
Overall	69% (n = 29)	49% (n = 33)				0.0328	0.0632
Sharers	50% (n = 4)	49% (n = 27)				0.6278	0.8188
***Location of Fishing Activity***	***Boat***	***Shore***					
Overall	80% (n = 39)	38% (n = 23)				<0.0001	0.0002
Sharers	79% (n = 19)	31% (n = 12)				<0.001	0.0008
***Education Level***	***Grades 1-9***	***Grades 10-12***	***College or Graduate School***				
Overall	30% (n = 6)	51% (n = 25)	77% (n = 30)			<0.001	*--*
Sharers	20% (n = 3)	46% (n = 13)	74% (n = 14)			0.0019	*--*
***Effect of Signage***	***Pre-Intervention***	***Post-Intervention***					
Overall	57% (n = 32)	57% (n = 30)				0.5988	0.6208
Sharers	53% (n = 20)	44% (n = 11)				0.8232	0.2013

* The reported *p*-values in this column correspond to one-sided Fisher’s Exact Test for consumption of fish, location of fishing activity, knowledge of the term “fish consumption advisory” and effect of signage or Exact Mantel-Haenszel Chi-Square Test for age and education level; ^†^ The reported *p*-values correspond to Mantel-Haenszel Chi-Square Test; ^††^ Parenthetical numbers indicate the number of participants in each category who had knowledge of the term “fish consumption advisory”.

**Table 3:** Group differences in knowledge of the fish consumption advisory for Badin Lake for the overall sample of study participants at Badin Lake, NC, USA compared to a subsample that shares fish caught with women and children.

Variable of Comparison	Knowledge by Comparison Groups					*p-*value *	*p-*value Controlling for Education ^†^
***Age (years)***	***18–24***	***25–34***	***35–44***	***45–54***	***55+***		
Overall	33% (n = 2) ^††^	26% (n = 7)	26% (n = 6)	36% (n = 9)	75% (n = 21)	<0.001	0.0006
Sharers	25% (n = 1)	18% (n = 3)	29% (n = 4)	33% (n = 4)	63% (n = 10)	0.0131	0.0112
***Consumption of Fish from Badin Lake***	***No***	***Yes***					
Overall	43% (n = 18)	40% (n = 27)				0.4734	0.9462
Sharers	25% (n = 2)	36% (n = 20)				0.8476	0.2657
***Location of Fishing Activity***	***Boat***	***Shore***					
Overall	71% (n = 35)	17% (n = 10)				<0.0001	<0.0001
Sharers	79% (n = 19)	8% (n = 3)				<0.0001	<0.0001
***Education Level***	***Grades 1–9***	***Grades 10–12***	***College or Graduate School***				
Overall	30% (n = 6)	41% (n = 20)	46% (n = 18)			0.2785	--
Sharers	13% (n = 2)	39% (n = 11)	42% (n = 8)			0.1058	--
***Knowledge of Term “Fish Consumption Advisory”***	***No***	***Yes***					
Overall	11% (n = 5)	65% (n = 40)				<0.0001	<0.0001
Sharers	9% (n = 3)	61% (n = 19)				<0.0001	0.0001
***Effect of Signage***	***Pre-Intervention***	***Post-Intervention***					
Overall	32% (n = 18)	51% (n = 27)				0.0359	0.0813
Sharers	32% (n = 12)	40% (n = 10)				0.3373	0.9316

* The reported *p*-values in this column correspond to one-sided Fisher’s Exact Test for consumption of fish, location of fishing activity, knowledge of the term “fish consumption advisory” and effect of signage or Exact Mantel-Haenszel Chi-Square Test for age and education level; ^†^ The reported *p*-values correspond to Mantel-Haenszel Chi-Square Test; ^††^ Parenthetical numbers indicate the number of participants in each category who had knowledge of the Badin Lake advisory.

Differences were found in knowledge of the term “fish consumption advisory” (*p* < 0.0001) and knowledge of the fish consumption advisory specifically for Badin Lake (*p* < 0.0001) between fishing locations (*i.e.*, shore or boat). Awareness was more prevalent among boat anglers in each case. Similarly, a significant difference (*p* < 0.001) was found in knowledge of the term “fish consumption advisory” among shore anglers who share fish with women and/or children and boat anglers who share fish with women and/or children; shore anglers (31%, n = 12) were less likely than boat anglers (79%, n = 19) to know this term. Shore anglers who share fish with women and children (8%, n = 3) were also significantly (*p* < 0.0001) less likely to be aware that there was a Badin Lake-specific fish consumption advisory than boat anglers (79%, n = 19). Significant differences between fishing location were observed for knowledge of the term (*p* = 0.0002) and for the Badin Lake advisory (*p* < 0.0001) after controlling for education level.

For the entire sample of anglers, there was a significant increase (*p* = 0.0359) in the percentage of anglers who were aware that a fish consumption advisory was in place for Badin Lake from pre-intervention (32%, n = 18) to post-intervention (51%, n = 27). The intervention had no significant effect on knowledge of the term “fish consumption advisory” for the overall sample or the sharing subsample. Knowledge of the Badin Lake advisory was significantly higher among anglers who knew the “fish consumption advisory” term for the overall angler sample (*p* < 0.0001) and sharers (*p* < 0.0001).

Before signs were posted at Badin Lake, 68% (n = 26) of anglers who share the fish they catch with women and/or children did not know that there was a fish consumption advisory for the lake. Following the posting of the signs, 60% (n = 15) did not know that there was a fish consumption advisory for the lake, indicating that there was no significant change in knowledge of the fish consumption advisory pre- and post-intervention among anglers who share with women and children. The majority of anglers who share their catch of fish from Badin Lake were not aware of a fish consumption advisory either before or after the intervention. It should be noted, however, that the percentage favors an increase in knowledge post-intervention, and the sample size may be too small to have detected any real change.

### 3.3. Angler Perceptions of Advisory Signs

Participant criticisms of the current Badin Lake fish consumption advisory sign, which was not designed using a research-based approach or involving the target audience, were that the sign was “not eye-catching”, “won’t catch my attention”, and “hard to read”. Suggestions for improvement (n = 19 respondents) included making the sign “bigger”, incorporating “more color”, and adding words such as “danger”, “warning”, “caution”, or “stop”. 

## 4. Discussion and Conclusions

### 4.1. Awareness Differences by Subpopulation

This study examined the effectiveness of a sign that was developed by the North Carolina Division of Public Health to communicate a newly issued fish consumption advisory for Badin Lake, a man-made reservoir on the Yadkin-Pee Dee River in central North Carolina, USA. Important differences in the knowledge of the fish consumption advisory were found among subpopulations of anglers. Generally, younger anglers, those who did not know the term “fish consumption advisory”, and shore anglers were observed to have lower levels of knowledge of the Badin Lake advisory. For the interviewees who participated in this study, knowledge of the fish consumption advisory for Badin Lake was found to be 32% before the posting of the sign and 51% after its posting, reflecting a significant increase in awareness. The literature reports variability in knowledge of fish consumption advisories among anglers fishing in other waters under advisories, from approximately 50% to more than 80% [15,16,17,18,19]. The utility of signs in communicating fish advisories likewise varies. For example, signs posted at boat launches, fishing sites, and other locations where anglers might frequent have been identified by fewer than 10% [16], approximately 20% [17,18], and more than 60% [19] of anglers as being a source of information about fish consumption advisories, depending on the fishing site and population studied.

Key findings from the present study include significant differences in awareness of the term “fish consumption advisory” and of the fish consumption advisory in effect for Badin Lake between shore and boat anglers. Shore anglers were significantly less likely to be aware of the term (*p* < 0.0001) or of the specific advisory (*p* < 0.0001) than boat anglers. Significant differences in knowledge by fishing location were observed after controlling for education. Although income frequently co-varies with education, this difference in awareness may be an artifact of income differences between boat and shore anglers, as significant differences in knowledge of fish consumption advisories have been found by income in previous studies, with lower income anglers’ exhibiting lower levels of awareness [15,16]. Other explanations for the observed differences include greater immersion in angling issues and culture among boat anglers and increased visibility of signs near boat launches. Boat and shore angler differences in awareness were found for the entire study sample and for the subsample of anglers who share fish with women and children. This finding has importance for state health departments and others who issue fish consumption advisories. Future educational efforts should specifically target shore anglers and seek more effective means of communicating with this subsample of the angling population.

An emerging issue in this study was the effectiveness of the advisory sign in protecting vulnerable women and children subpopulations for whom more restrictive limits on fish consumption have been issued. Although a significant (*p* = 0.0359) increase in knowledge of the fish consumption advisory was found for the entire sample of study participants, a commensurate increase in knowledge was not found for the subsample of anglers who share their catch with women and children. The subsample of anglers who share their catch with women and children had a small increase (32% to 40%) in knowledge of the advisory at Badin Lake, which was not statistically significant. The sign evaluated in this study was less effective in communicating advisory information to anglers, in this case the male primary informants, who possibly provide fish to the most sensitive subpopulations. 

Several studies have examined the knowledge of fish consumption advisories for women as fish consumers [20,21,22]. Little attention has been paid to male angler knowledge of, and compliance with, fish consumption advisories as the first line of defense against consumption of fish that is restricted for specific subpopulations. One notable exception is work by Burger *et al.* [23], who evaluated the utility of a brochure designed for pregnant women, women with small children, and women who plan to become pregnant in increasing anglers’ knowledge of messages related to fish consumption advisories for women of childbearing age and children. In their study, anglers had lower levels of understanding of these key messages than the women for whom the materials were originally developed, although the brochure was generally effective in informing anglers. These findings, in conjunction with the findings from the present study, suggest the need for improved communication materials for anglers who potentially share their catch with sensitive subpopulations.

Although this study identifies priority areas for health departments and those who issue fish consumption advisories to create more targeted fish consumption advisory communication strategies, the study is limited by the scope of the data collected, the use of convenience samples, and the administration of the questionnaire in English and Spanish only. Data regarding fish species caught and consumed, fishing and eating behaviors, characteristics of women and children with whom the subsample of interest shared their catch, and other possible sources of information available to anglers regarding the advisory were not collected during this study. To an extent, the scope of the questionnaire was limited by a commitment on the part of the Division of Public Health to minimize intrusiveness in order to maximize participation. Further, these limitations reflect the reality of fish consumption advisory communication and evaluation approaches employed by state and local health departments. 

### 4.2. Policy Implications

From the study findings, specifically the findings highlighting differences in fish consumption advisory signage effectiveness among subsamples, one can conclude that state and local health departments and others effecting policies and messages related to fish consumption advisories should consider the communication needs of multiple target audiences when issuing and conveying these advisories. Efforts to communicate these advisories to sensitive subpopulations and the individuals who potentially influence contaminant exposure in those sensitive subpopulations (e.g., the largely male anglers, primary informants, in this study who share their catch with women and children) should be a priority for both levels of government. Examining this key subsample of anglers who possibly share their catch with sensitive subpopulations, apart from anglers overall, proved to be useful and allowed for the identification of differences in sign efficacy. This observation resonates with a recent position paper by Gochfeld and Burger [24] that advocates for greater awareness of, and protections for, high-risk subpopulations, including children, in environmental health, exposure, and risk assessment despite their outlier status.

As has been found in previous studies [17,18,19], signs for fish consumption advisories were found to be a useful tool in communicating fish consumption advisories to the overall population of anglers. This study, however, suggests that the current sign used at Badin Lake may not be as effective in conveying advisories to shore anglers and anglers who share their catch with women and children and may thus not be effectual in protecting the most sensitive subpopulations for whom the fish consumption advisory is most restrictive. Future research should explore more efficacious means of communicating with these subsamples. 

### 4.3. Future Research

Future studies should involve anglers, and specifically anglers who share their catch with women and children and shore anglers, in developing communication materials, including improved signage, that address participants’ criticisms and suggestions for improvement. Subsequent research should also address limitations of this current study, including the collection of more extensive demographic information and comprehensive data related to angler intent to change behaviors related to advisory messages. Anglers’ intention to change behavior in alignment with advisories and current compliance with advisories have been found to be markedly lower than awareness of the advisories [17,18] and will be a focus of future efforts. Our preliminary data suggest that behavior modification among anglers at Badin Lake is limited as a result of the issuance of the advisory and the signage intervention. With a reported trend of exponential increases in the issuance of fish consumption advisories in the U.S. [12], the communication of advisories to anglers, and specifically to anglers who share their catch with women and children, will have growing significance for public health in general and maternal and child health in particular.
